# Attention-guided erasing for enhanced transfer learning in breast abnormality classification

**DOI:** 10.1007/s11548-024-03317-6

**Published:** 2025-01-15

**Authors:** Adarsh Bhandary Panambur, Sheethal Bhat, Hui Yu, Prathmesh Madhu, Siming Bayer, Andreas Maier

**Affiliations:** 1 Siemens Healthineers, Karl Heinz Kaske Str. 5, 91052 Erlangen, Bayern Germany; 2 Pattern Recognition Lab, Friedrich-Alexander-University Erlangen-Nuremberg, Martensstr. 3, 91058 Erlangen, Bayern Germany

**Keywords:** Breast cancer, Mammography, Data augmentation, Self-supervised learning, Transfer learning

## Abstract

**Purpose:**

Breast cancer remains one of the most prevalent cancers globally, necessitating effective early screening and diagnosis. This study investigates the effectiveness and generalizability of our recently proposed data augmentation technique, attention-guided erasing (AGE), across various transfer learning classification tasks for breast abnormality classification in mammography.

**Methods:**

AGE utilizes attention head visualizations from DINO self-supervised pretraining to weakly localize regions of interest (ROI) in images. These localizations are then used to stochastically erase non-essential background information from training images during transfer learning. Our research evaluates AGE across two image-level and three patch-level classification tasks. The image-level tasks involve breast density categorization in digital mammography (DM) and malignancy classification in contrast-enhanced mammography (CEM). Patch-level tasks include classifying calcifications and masses in scanned film mammography (SFM), as well as malignancy classification of ROIs in CEM.

**Results:**

AGE significantly boosts classification performance with statistically significant improvements in mean F1-scores across four tasks compared to baselines. Specifically, for image-level classification of breast density in DM and malignancy in CEM, we achieve gains of 2% and 1.5%, respectively. Additionally, for patch-level classification of calcifications in SFM and CEM ROIs, gains of 0.4% and 0.6% are observed, respectively. However, marginal improvement is noted in the mass classification task, indicating the necessity for further optimization in tasks where critical features may be obscured by erasing techniques.

**Conclusion:**

Our findings underscore the potential of AGE, a dataset- and task-specific augmentation strategy powered by self-supervised learning, to enhance the downstream classification performance of DL models, particularly involving ViTs, in medical imaging.

## Introduction

Recent estimates indicate that breast cancer ranks as the leading cause of female cancer incidence worldwide, with approximately 2.3 million cases reported globally, thus representing a significant healthcare burden [1]. Digital mammography (DM) stands out as the predominant screening modality employed for breast cancer screening in patients. During initial screenings, radiologists typically employ the Breast Imaging Reporting and Database System (BI-RADS) scoring system [2] to note various indications in the breast, including the assessment of breast density and potential abnormalities. Breast density refers to the proportion of fibroglandular tissue present in the breast. Numerous studies have established correlations between increased breast density and elevated breast cancer risk [3]. Furthermore, clinicians assign a BI-RADS score to each patient, aiding in the determination of appropriate periodic screening protocols based on the presence of abnormalities such as masses and calcifications [2]. Following DM screening, additional imaging modalities might be recommended for certain patients, particularly those with dense breast tissue. Notably, in Asian populations, women often exhibit denser breast tissues, impacting mammographic screening sensitivities [4]. Contrast-enhanced mammography (CEM) has emerged as a valuable tool for providing supplementary diagnostic information in such cases [5].

However, with an increasing population eligible for screening worldwide, it becomes imperative to streamline radiologists’ workflows. In this context, computer-aided diagnosis tools supported by deep learning (DL) have proven beneficial, particularly in assisting radiologists by reducing diagnostic errors, such as false positives and false negatives and by providing a DL-based second opinion, in turn leading to enhanced screening sensitivities [6]. Several studies have investigated the application of DL algorithms for classification tasks within breast cancer screening and diagnostic workflows, particularly in DM and CEM [6–10]. Nevertheless, the design of robust deep-learning algorithms necessitates high-quality annotations, which are both time-consuming and expensive to obtain. Recent research has highlighted the advantages of self-supervised learning (SSL) algorithms trained using vision transformers (ViTs) [11, 12]. Notably, these algorithms possess weak localization capabilities, eliminating the need for class labels during training. Methods like DINO have demonstrated robust representation learning capabilities [11]. Some studies have explored the weak localization properties of DINO in medical imaging, particularly in breast imaging analysis using DM [13]. In such analysis, properties of weak localization of region of interest (ROI) such as dense tissue in breast density classification are explored, rendering these algorithms advantageous for downstream classification [13].

In this study, we evaluate the effectiveness and generalizability of our recently proposed data augmentation technique, named attention-guided erasing (AGE) [13], for various tasks in breast abnormality classification across different mammographic imaging modalities. AGE employs attention head visualizations from a ViT model, that is trained using the DINO SSL approach [11]. These visualizations weakly localize different regions within the breast tissue in mammography images. The weak localizations are then used to selectively remove background regions during training with a random probability to emphasize only the regions of interest for further analysis by the network. Initially proposed for breast density classification [13], this research aims to assess the benefits and limitations of AGE, focusing on its generalizability across different scenarios with publicly available datasets for tasks based on the presence of unique regions of interest. The main contributions of our work include: (a) Demonstrating the robustness of our proposed AGE augmentation by achieving statistically significant results across three different datasets for four different tasks in mammography. (b) Providing a comprehensive quantitative and qualitative analysis to discuss the pros and cons of AGE augmentation.

## Methods

**Fig. 1:**
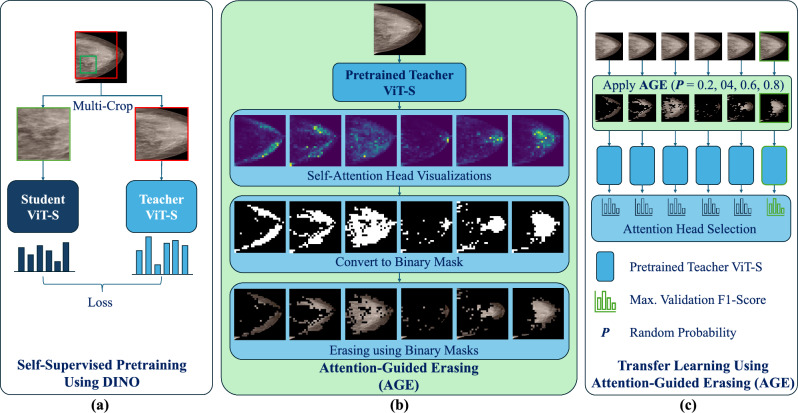
Overview of the Attention-Guided Erasing (AGE) Methodology. **a** Self-Supervised Pretraining using DINO [11]: A teacher student ViT framework, leveraging a teacher-student ViT-S self-distillation framework. **b** AGE [13]: Attention head visualizations from the SSL pretrained teacher ViT-S are converted into binary masks to isolate key ROIs and then used to erase background regions. **c** Transfer Learning with AGE: AGE is used on the input images using each of the attention heads with a random probability during training. The attention head yielding the highest validation performance is selected for final AGE-based transfer learning

Figure 1 outlines our proposed AGE data augmentation pipeline, involving SSL pretraining using DINO [11], a step-by-step illustration of obtaining masks for AGE, and transfer learning using AGE [13]. A single mammogram image is used in this figure for illustrative purposes, while the methodology is applied across multiple datasets as detailed in this work. The details of each stage are provided in this section.

### Self-Supervised pretraining using DINO

Figure 1(a) shows the overview of the DINO SSL pretraining. DINO’s approach involves a self-knowledge distillation paradigm where the student network learns to replicate the output of a teacher network through cross-entropy loss, aligning their output probabilities [11]. The output probabilities are derived by applying a softmax function to the feature representations learned independently by the student and teacher networks. Two differently transformed versions of the same image acts as the input to the student and teacher networks, which have distinct parameters. Here, we adopt the original implementation of DINO [11], to apply a multi-crop strategy, utilizing two global crops of the mammograms along with multiple lower-resolution local crops that focus on smaller patches within the images. The exposure to varied transformations drives the SSL learning process [11]. To maintain the quality of the model, the teacher network’s parameters are gradually updated to reflect the student network’s parameters, using an exponential moving average [11]. The ViT architecture is employed for both student and teacher networks, where the input image is split into patches, embedded, and an additional learnable class token (CLS) without labels is included to aggregate information [11]. These embeddings are processed through self-attention layers to capture relationships between patches.

### Attention-guided erasing

Figure 1(b) shows the step-by-step illustration of the AGE data augmentation. After the SSL pretraining, the self-attention mechanisms are carefully analyzed in the final layer’s six attention heads, each focusing on the CLS token [11]. As seen in Fig. 1(b), the attention maps generated by these heads are converted into binary masks through thresholding. These binary masks are used to erase non-attention regions in the image, retaining only the regions of interest to perform AGE [13].

### Transfer learning using AGE

Examining the activation patterns that most accurately represent the feature of interest across the datasets is crucial. In the AGE method for breast density classification [13], we manually analyzed the activation patterns of each of the six attention heads to understand their relevance to breast tissue characteristics. We then hypothesized that an attention head focusing on fewer than 50 pixels could be identifying regions of dense breast tissue. To test this hypothesis, we analyzed these activation patterns in 10% of our training data set and found that the sixth attention head consistently concentrated on dense areas, suggesting its potential association with breast density [13]. However, for tasks requiring expert domain knowledge, such as abnormality or malignancy classification explored in this work, selecting the optimal attention head that accurately localizes the ROI presents a challenge. Figure 1(c) shows the depiction of the attention head selection during transfer learning using AGE. We treat each attention head as a hyperparameter and evaluate the performance of AGE across six separate runs, using binary masks generated from each of the six attention heads with a random probability during training. The attention head achieving the highest validation F1-score across these runs is selected as the optimal head and used for the final application of AGE. We adopt this selection approach because the imaging presentation of malignancy, such as calcifications or masses, is inherently more complex and less distinct compared to breast density. Additionally, acquiring domain-specific knowledge in the form of pixel-level annotations is generally time-consuming and costly, further complicating the process.

### Dataset

In our research, we utilize four distinct datasets consisting of different dataset sizes to efficiently evaluate the AGE augmentation technique: VinDr-Mammo [14], CDD-CESM [15], and two subsets derived from the CBIS-DDSM dataset [16]. These datasets are selected based on the diversity of mammographic modalities they represent. This ensures a broad variety in how the input images manifest the features essential for the image-level and patch-level classification tasks. The aim is to validate the generalizability of our technique by thoroughly assessing the applicability and performance across different image classification tasks.

#### Breast density classification

The VinDr-Mammo dataset, which originates from Hospital 108 and Hanoi Medical University Hospital in Hanoi, Vietnam, comprising 20,000 full-field DM from 5,000 patients was utilized for the image-level breast density (A, B, C, D) classification task [14]. The ROI, i.e., dense tissue, appears in varying proportions within the DM image and serves as the input to the DL system. We use this dataset to discuss the results originally published in [13]. It includes both craniocaudal and mediolateral oblique views, thoroughly annotated by expert radiologists for BI-RADS breast density. The dataset was divided into training (72 A, 1,308 B, 10,366 C, 1,854 D), validation (8 A, 220 B, 1,866 C, 306 D), and testing (20 A, 380 B, 3,060 C, 540 D) portions.

#### Malignancy classification

The subtracted CEM images from the CDD-CESM dataset are utilized for both image-level and patch-level classification tasks [15]. Various abnormalities appear throughout the image in varying size proportions and lesions are visually enhanced due to the contrast agents used while acquiring the images. The dataset comprises 1,003 CEM images from Cairo University, Egypt, stratified at the patient level into training (75%: 309 normal, 193 benign, 243 malignant), validation (13%: 61 normal, 34 benign, 35 malignant), and testing (12%: 46 normal, 23 benign, 47 malignant) sets [15].

#### Calcification classification

The calcification subset of CBIS-DDSM containing ROIs in digitized scanned film mammography (SFM) is then used as the distributions, morphology, and intensities of the calcifications in the dataset vary in proportion to the input ROI image [17]. For the calcification abnormality subset of CBIS-DDSM, the distribution is as follows: training (389 benign without callback, 438 benign, 446 malignant), validation (85 benign without callback, 90 benign, 98 malignant), and testing (67 benign without callback, 130 benign, 129 malignant) [16, 17].

#### Mass classification

The CBIS-DDSM data subset consisting of mass abnormality is utilized here, where the shape and presence of masses encompass the entire input ROI patch [16]. The mass abnormality subset is similarly divided, for training (91 benign without a callback, 468 benign, 525 malignant), validation (13 benign without a callback, 109 benign, 112 malignant), and testing (37 benign without a callback, 194 benign, 147 malignant).

### Experimental setup

For the SSL pretraining, the original DINO implementation [11] is used for each task, where the ImageNet pretrained ViT-S architecture is used as the backbone for both the student and teacher networks [11].The standard data augmentation techniques reported in the original work such as flipping, color adjustments, blurring, and solarization are reused [11]. The input images are subjected to random resizing and cropping to match the model’s input specifications, with a patch size of 16 for the 224 $\times$ 224 resolution images. Pretraining is performed with a batch size of 32, suitable for the memory capacity of a single GPU over 300 epochs, and the models are saved at the lowest loss points. We compare our AGE augmentation with a standard random erasing (RE) [18] augmentation during the downstream transfer learning task at various probability values (*P*) of 0.2, 0.4, 0.6, and 0.8. The probability value indicates the chance that an image will undergo augmentation during training. A classification layer on top of the pretrained teacher ViT-S model for all classification tasks is added and the entire network is finetuned during the transfer learning. All experiments are conducted five times over 100 epochs with a batch size of 8, incorporating early stopping to prevent overfitting. Optimization was performed using a binary cross-entropy loss and a standard Adam optimizer, with a learning rate of $5e^{-6}$ and a weight decay of $1e^{-4}$. A standard set of data augmentations is utilized, as reported in [19].

**Table 1:** Classification performance for image-level tasks on the test datasets with their respective sizes in brackets. The best results are in **bold**. Asterisks indicate the statistical significance of AGE over the NE and RE, $p < 0.0001$

Dataset	VinDR-Mammo	CDD-CESM
(Test)	(4000)	(116)
No Erasing	$0.5594 \pm 0.026$	$0.6076 \pm 0.045$
Random Erasing	$0.5691 \pm 0.020$	$0.6406 \pm 0.048$
**Attention-Guided Erasing**	$\mathbf {0.5910 \pm 0.017}^{\tiny {*}}$	$\mathbf {0.6579 \pm 0.010}^{\tiny {*}}$

**Table 2:** Classification performance for patch-level tasks on the test datasets with their respective sizes in brackets. The best results are in **bold**. Asterisks indicate the statistical significance of AGE over the NE and RE, $p < 0.0001$, and the plus sign indicates significance with $p<0.05$

Dataset	CBIS-DDSM (Calc)	CDD-CESM	CBIS-DDSM (Mass)
(Test)	(326)	(116)	(378)
No Erasing	$0.6803 \pm 0.016$	$0.7617 \pm 0.023$	$0.5530 \pm 0.007$
Random Erasing	$0.6846 \pm 0.017$	$0.7859 \pm 0.012$	$0.5471 \pm 0.014$
**Attention-Guided Erasing**	$\mathbf {0.6892 \pm 0.007}^{\tiny {*}}$	$\mathbf {0.7920 \pm 0.026}^{\tiny {+}}$	$\mathbf {0.5537 \pm 0.016}$

## Results and discussion

**Fig. 2:**
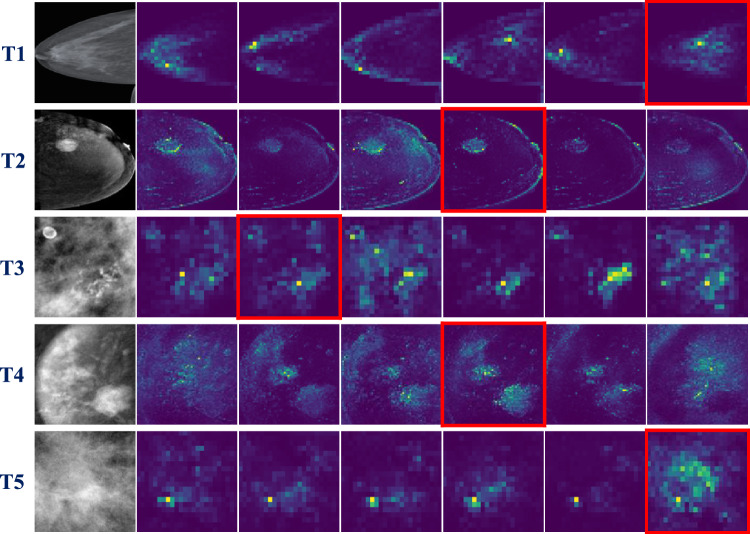
**Attention Head Visualizations.** Input image followed by six attention maps from each of the five pretrained DINO models associated with specific tasks: T1 (Breast Density in DM), T2 (Malignancy in CEM), T3 (Calcification ROI in DM), T4 (Malignancy ROI in CEM), and T5 (Mass ROI in DM). The final selected attention heads used for transfer learning are highlighted in red

**Fig. 3:**
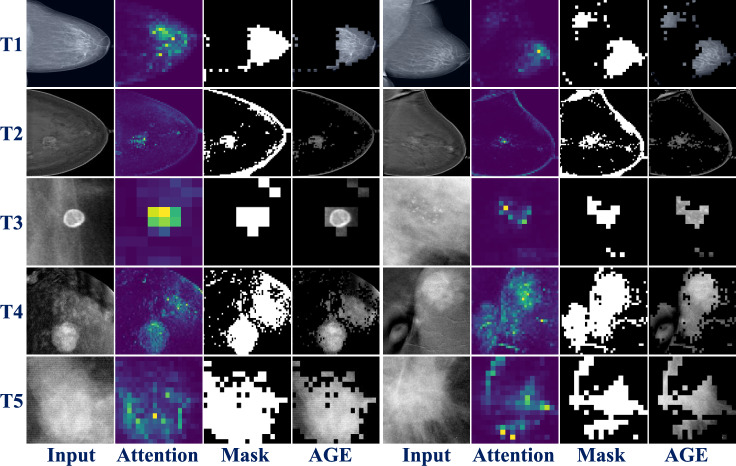
**Qualitative Examples of AGE.** Images (Input) from each classification task: T1 (Breast Density in DM), T2 (Malignancy in CEM), T3 (Calcification ROI in DM), T4 (Malignancy ROI in CEM), and T5 (Mass ROI in DM), the final selected attention head (Attention), the corresponding binary mask (Mask), and the resulting erasure applied to the image (AGE)

Tables 1 and 2 present a comprehensive overview of the quantitative classification performance on test datasets for the image-level and patch-level classification tasks, respectively. We utilize the mean macro F1-scores with associated standard deviations from five runs to evaluate the classification performance to mitigate the variability in the class sample distribution across the different tasks. The performances are evaluated under three different conditions: no erasing (NE), random erasing (RE), and attention-guided erasing (AGE) (Tab. 1 and Tab. 2). We present the best results arising from different probability values (*P*) used for the augmentations during training as mentioned in Sect. "Experimental setup". Figure 2 shows the visual attention maps from the six different attention heads of five different pretrained DINO models T1-T5 for each of the datasets utilized in this work. The final attention head selected for each of the tasks are marked in red.Figure 3 presents qualitative examples of the AGE methodology, including images from each classification task (T1-T5), the final selected attention head (Attention), the corresponding binary mask (Mask), and the resulting erasure applied to the image (AGE).

For the image-level breast density categorization task, as seen in the T1 row of Fig. 2, the sixth attention head mostly focuses on the dense breast tissue and therefore is used for performing AGE [13]. The application of AGE (*P* = 0.6) augmentation resulted in the highest mean macro F1-score of 0.5910 ± 0.017, indicating a significant improvement over the NE with 0.5594 ± 0.026 and RE (*P* = 0.2) with 0.5691 ± 0.020 augmentations [13]. Upon performing a two-tailed unpaired t-test to assess the statistical significance, we achieved a p-value of $p<0.0001$ between AGE to both NE and RE augmentations, as seen in [13]. As per the BI-RADS definition, we know the appearance of dense breast tissue concerning the whole input image increases in proportions starting from density A (entirely fatty) to density D (extremely dense) [2]. Therefore it can be implied that applying AGE has a positive impact as it forces the network to focus more on this ROI rather than on the larger proportions of input containing the irrelevant background parts, as depicted in T1 row of Fig. 3 [13].In this row, we observe that in the CC and MLO views, AGE is able to effectively retain the dense breast tissue. However, in the MLO view, a small amount of attention is also retained over the pectoralis muscle. To investigate this further, we conducted experiments using CC and MLO view classifiers; however, no statistically significant differences were observed. This could be attributed to the higher proportion of dense breast tissue in the Vietnamese cohort, where the overall dense appearance of the images might overshadow the diagnostic contribution of high-intensity regions.As the size of this dataset is substantially larger in comparison with the other datasets used in this research and the dataset is also highly imbalanced, the performance gains are larger in this case. The next image-level classification task involves the classification of full CEM images into normal, benign, and malignant classes. The attention head visualizations of this task can be seen in the T2 row of Fig. 2. We can observe that multiple attention heads weakly localize the various regions of interest, with especially the fourth attention head concentrating on the multiple areas of the CEM. The T2 row of Fig. 3 shows some examples of AGE retaining the malignant regions in the CC and MLO views and erasing the backgrounds. When applying AGE augmentation (*P*=0.4) using the fourth attention head, we achieve statistically significant improvements with a mean F1-score of $0.6579 \pm 0.010$, over both NE with $0.6076 \pm 0.045$ and RE (*P*=0.6) with $0.6406 \pm 0.048$, with a p-value of $p<0.0001$ (Tab. 1).

In mammography, patch-level classification might lead to finer granularity in identifying subtle abnormalities by analyzing smaller regions within the image, compared to image-level classification, due to the higher resolutions of mammograms. To observe the generalizability of AGE in such scenarios, extensive experimentation is conducted across three different ROI patch-level classification datasets. The attention head visualizations for the calcification subset dataset are presented in the T3 row of Fig. 2. Notably, the second attention head focuses on the calcification regions in the input images, which appear in two distinct areas of the image (top left and bottom). This pattern is consistent throughout the dataset and upon an initial hyperparameter search for the attention head selection, is utilized for applying AGE. We demonstrate a notable improvement, with the best performance scored at 0.6892 ± 0.007 using AGE (*P*=0.2), compared to NE with 0.6803 ± 0.016 and RE (*P*=0.6) with 0.6846 ± 0.017 (Table 2). The statistical significance of this improvement is confirmed with a *p*-value of less than $p<0.0001$ when comparing AGE with respect to both NE and RE. As detailed in Sect. "Calcification classification", the dataset is fairly balanced and comparatively very small. Even in this scenario, AGE positively impacts the classification performance by guiding the network’s attention toward the high-intensity regions of calcifications in the input image by focusing on features such as the high-intensity spot and distribution of the calcifications as seen in T3 row of Fig. 3.For CEM ROI image classification, the attention head visualizations can be seen in the T4 row in Fig. 2. As observed, the fourth attention head focuses mostly on the class-specific features. When applying AGE (*P*=0.2) using the fourth attention head, we observe a substantial improvement when using AGE, with a mean macro F1-score of 0.7920 ± 0.026. Compared to the NE and RE (*P*=0.2), with mean F1-scores of 0.7617 ± 0.023 and 0.7859 ± 0.012, AGE significantly improves classification performance, with a *p*-value of 0.0227, indicating its effectiveness in differentiating between normal, benign, and malignant classes in CEM (Table 2). Moreover, as observed in Sect. "Malignancy classification", the dataset is the smallest among the ones used in this research and still outperforms the baselines. The T4 row in Fig. 3 shows two examples of AGE applied on malignant lesions.

The attention head visualizations and AGE applied onmass abnormality can be observed in the T5 row of Fig. 2 and Fig. 3, respectively.As observed in the input image, the breast mass appears as a dense region in the ROI images encompassing the entire resolution of the input ROI. When utilizing the AGE augmentation (*P*=0.6) using the sixth attention head, we show the performance of 0.5537 ± 0.016, where the improvement over the NE with 0.5530 ± 0.007 is marginal (Table 2). We further observe that RE (*P*=0.6) works detrimentally with a performance drop only in the mass analysis task in comparison with the other tasks. This shows the shape and margins of the mass are important features that are not preserved by using RE augmentation. However, the AGE augmentation slightly improves upon the baseline showing that our approach does not degrade the performance in tasks where the ROI covers the entire input image.

## Conclusion and outlook

In conclusion, we validate our recently proposed novel data augmentation technique named AGE for enhancing breast abnormality classification during transfer learning. The generalizability and effectiveness of AGE are systematically assessed across a diverse set of tasks and datasets, demonstrating statistically significant improvements in classification performance evaluated with mean F1-scores compared to traditional techniques, for both image-level and patch-level classification tasks. Notably, for image-level classification tasks concerning breast density in DM and malignancy classification in CEM, advancements of 2% and 1.5% are attained, respectively. Moreover, in the domain of patch-level classification targeting ROIs of calcifications in SFM and ROIs of lesions in CEM, improvements of 0.4% and 0.6% are, respectively, observed. The technique’s ability to focus the model’s attention on relevant regions of interest while discarding non-essential background information has proven instrumental in achieving these advancements. As highlighted in the literature where data augmentation techniques have significantly enhanced mammography analysis in DL [20], AGE, combined with the SSL pretrained backbone, improves model performance and reliability. Using interpretable attention heads, it can potentially aid radiologists by highlighting critical regions, reducing diagnostic complexity, and streamlined the analysis of imaging features. However, our work is based on retrospective datasets, which may limit the generalizability of the findings to real-world clinical settings. Moreover, the marginal improvement observed in the mass classification task underscores the necessity for further research to refine the application of augmentation techniques in scenarios where critical features might be inadvertently obscured. This highlights that prior clinical knowledge might be crucial for effectively applying AGE, particularly in tasks where critical features span the entire image, to optimize its clinical utility.

The distinction in performance across different tasks highlights the importance of a contextually adaptive augmentation strategy which can be dataset-specific to maximize the potential of ViT models in mammographic analysis. This research not only contributes to the advancement of mammography analysis but also sets a precedent for the investigation of attention-based data augmentation techniques in other medical imaging modalities. Future directions include analyzing mammographic view-based differences in tasks like BI-RADS categorization to align with clinical insights, broadening the AGE method to diverse medical imaging domains, refining the selection method of attention heads, and integrating AGE with other SSL frameworks that offer superior localization [12]. Furthermore, in alignment with recent studies emphasizing the creation of dataset-specific loss functions for improved downstream classification outcomes [21], we plan to incorporate such loss functions in conjunction with AGE to further enhance the downstream classification performance. Our findings demonstrate the critical role of carefully designed data-specific solutions which can significantly enhance the performance of DL models.

## Data Availability

We utilized publicly available open-access datasets for our experiments and extend our gratitude to the authors and institutions behind VinDr-Mammo, CDD-CESM, CBIS-DDSM, and ‘The Cancer Imaging Archive’ (TCIA) [4, 15–17].

## Declarations

### Disclaimer.

The methods in this paper are not available for commercial use, with no guarantee of future availability. Intended for research use only.
